# Subclinical Atherosclerosis Could Increase the Risk of Hearing Impairment in Males: A Community-Based Cross-Sectional Survey of the Kailuan Study

**DOI:** 10.3389/fnins.2022.813628

**Published:** 2022-04-25

**Authors:** Chunyu Ruan, Xiang Mao, Shuohua Chen, Shouling Wu, Wei Wang

**Affiliations:** ^1^Department of Cardiology, Kailuan General Hospital, Tangshan, China; ^2^Department of Otorhinolaryngology Head and Neck Surgery, Tianjin First Central Hospital, Tianjin, China; ^3^Institute of Otolaryngology of Tianjin, Tianjin, China; ^4^Key Laboratory of Auditory Speech and Balance Medicine, Tianjin, China; ^5^Key Medical Discipline of Tianjin (Otolaryngology), Tianjin, China; ^6^Otolaryngology Clinical Quality Control Centre, Tianjin, China

**Keywords:** subclinical atherosclerosis, hearing impairment, baPWV, PTA, cross-sectional survey

## Abstract

**Objective:**

The relationship between subclinical atherosclerosis and hearing impairment (HI) has not been widely considered. Brachial ankle pulse wave velocity (baPWV) is a good indicator of muscular artery elasticity and could be a feasible method to screen for subclinical atherosclerosis. Our study aimed to elucidate the relationship between baPWV and HI.

**Methods:**

This cross-sectional study was based on the Kailuan cohort. All participants completed a standardized questionnaire and underwent physical examinations and laboratory assessments at recruitment. Since 2010, some participants received additional baPWV testing during follow-up visits, and some who were exposed to occupational hazards such as noise received a pure-tone average hearing threshold (PTA) test after 2014. Male subjects with a complete physical examination, baPWV, and PTA data were recruited for this study. HI was defined as PTA > 25 dB. Multivariate linear and multivariate logistic regression analyses were used to evaluate the relationship between baPWV and PTA or HI.

**Results:**

Among 11,141 subjects, the age range was 18–65 years, with mean age of 43.3 ± 8.9 years, the average PTA was 20.54 ± 10.40 dB, and the detection rate of HI was 1,821/11,141 (16.3%). Subjects were divided into four subgroups according to baPWV quartile. As the baPWV quartile increased, age, systolic blood pressure, diastolic blood pressure, body mass index, total cholesterol, high-density-lipoprotein cholesterol, fasting blood glucose, PTA, and proportions of subjects reporting smoking, alcohol consumption, hypertension, and diabetes increased significantly (*p* < 0.05 for trend). The odds of HI were higher in the fourth quartile group [adjusted odds ratio (aOR): 1.33, 95% CI: 1.10–1.62] than in the first quartile group. For every 100 m/s increase in baPWV, the PTA increased by 13 dB (95% CI: 4–23). When we divided the subjects into young (5,478 subjects; age range 22–44 years; mean age 35.6 ± 5.5 years) or non-young subgroups (5,663 subjects; age range 45–65 years; mean age 50.7 ± 3.7 years) based on a cut-off age of 45 years, the aOR of the fourth quartile group increased to 2.65 (95% CI: 1.68–4.19), and the PTA increment increased to 18 dB (95% CI: 10–27) for every 100 m/s increase in baPWV in the young subgroup. However, this relationship became statistically insignificant in the non-young subgroup.

**Conclusion:**

Our study revealed the quantitative relationship between baPWV and HI in the Kailuan cohort subjects, although the results are not universally consistent in different populations.

## Introduction

Hearing impairment (HI) is a common public health concern around the world. Currently, the number of people with HI is approximately 466 million, and this number will exceed 900 million by 2050 (WHO, 2021). People with HI report problems in understanding speech, particularly in noisy listening conditions. Some studies have demonstrated a relationship between HI and depression or cognitive impairment (Lin et al., 2013; Wayne and Johnsrude, 2015), which burdens society and families. Once HI is established, there is no treatment to restore hearing, suggesting that identifying more potential modifiable risk factors of HI using simple but efficient screening methods to identify vulnerable populations and provide targeted primary prevention is essential.

Numerous risk factors that can induce HI alone or in combination have been widely acknowledged, including aging, genetic factors, noise exposure, and ototoxic drugs (Kenneson et al., 2002; Daniel, 2007; Corvino et al., 2018; Lieu et al., 2020). However, compared with these factors, the relationship between atherosclerosis and HI seems not to have been widely considered, especially in people with subclinical atherosclerosis. Atherosclerosis is a disease in which atherosclerotic plaques deposit in the walls of blood vessels and cause arteries to narrow. Several studies have tried to explain the pathomechanism that atherosclerosis increases the risk of HI (Hull and Kerschen, 2010; Helzner et al., 2011; Fischer et al., 2015), although individuals may not have noticeable symptoms such as hypertension, coronary heart disease, or stroke. Stria vascularis, which generates endolymphatic and endocochlear potential (EP), provides essential high potassium concentration condition for the mechanoelectric conversion process of ciliary cells. Stria vascularis is a highly vascularized cochlear region supplied by labyrinth arteries without collateral circulation and very sensitive to the impact of ischemic injury (Seidman et al., 1999; Tzourio et al., 2014). Arteriosclerosis reduces the blood flow of the labyrinth arteries to the stria vascularis; inadequate blood and oxygen supply blocks the adenosine triphosphate (ATP) utilization of Na^+^-K^+^-ATP enzyme in the stria vascularis, so that the EP could not be maintained and dropped significantly (Wangemann, 2002). The decrease of EP leads to ciliary cell death and decreased hearing sensitivity (Pirodda et al., 2016). The apical parts in the cochlea are the most vulnerable to ischemia, where the blood supply is most distal and low-frequency sound is transmitted (Frederiksen et al., 2014). Theoretically, arteriosclerosis initially affects lower frequencies (0.25, 0.5, and 1 KHz) (Liew et al., 2007). However, as the blood flow of the labyrinth arteries to the stria vascularis continues to be reduced, the stria vascularis from the top to the bottom of the cochlea will involve ischemia and the hair cells would undergo apoptosis, so that the HI would cover the whole frequency range. Therefore, early screening for subclinical atherosclerosis could be an effective method for HI detection.

The velocity at which the pulse wave travels through the artery wall is called pulse wave velocity (PWV), and it is considered a good indicator of atherosclerosis, marker of vascular injury, and clinical predictor of vascular disease (Pearson, 2002). The carotid to femoral PWV (cfPWV) is the gold standard for diagnosing arteriosclerosis, but the operation is complex, and cfPWV only reflects the elasticity of the aorta (Townsend et al., 2015; Williams et al., 2018). Brachial ankle PWV (baPWV) is a good substitute indicator of cfPWV that reflects the elasticity of the muscular arteries, including the aorta and the peripheral artery, and it is easy to measure and reproduce (Tanaka et al., 2009). Although baPWV could be a feasible method to screen for subclinical atherosclerosis, the relationship between baPWV and HI or pure-tone average hearing threshold (PTA) has not been consistently demonstrated (Yoshioka et al., 2010; Helzner et al., 2011; Chung et al., 2016), as there are only limited studies with large sample sizes or detailed demographic or health examination data useful for calculating the reliable parameter relationship.

The Kailuan study is a large community-based cohort of cardiovascular disease patients. This present cross-sectional survey based on the Kailuan study aimed to determine the reliable parameter relationship between baPWV and HI or PTA and explore the robustness after adjustment for demographic or physical condition confounders.

## Materials and Methods

### Study Participants

This cross-sectional study was based on a subset of the Kailuan cohort, an ongoing multicenter cohort in the study conducted in 2006–2007 and including 101,510 employees and retirees of a coal mining company (Kailuan Group Company) living in Tangshan City, China, with participant age ranging from 18 to 98 years. In the Kailuan cohort, all participants completed a standardized questionnaire and underwent physical examinations and laboratory assessments at recruitment in 11 hospitals (referred to hereafter as centers) responsible for community healthcare. The study participants were repeatedly and prospectively examined at 2-year intervals. Since 2010, some of the participants also received an additional baPWV test during the physical examination. Moreover, since 2014, the Occupational Disease Control Center of the Kailuan Group conducted a PTA test on subjects exposed to occupational hazards such as noise. The subjects who self-reported ear diseases, including otitis media, vestibular dysfunction, and sudden deafness, at baseline and follow-up in 2014–2015 were excluded. A total of 11,141 male subjects with a complete physical examination, baPWV and PTA data, and without head trauma or history of stroke were recruited for this study.

### Data Collection

Sociodemographic characteristics, lifestyle behaviors (smoking status, alcohol consumption, and physical activity), and medical history information were collected using questionnaires at baseline. Weight and height data of the subjects were collected, and the body mass index (BMI) was defined as weight (kg)/height (m)^2^. Blood pressure was measured using an HEN-8102A electronic sphygmomanometer (Omron Co., Ltd., Dalian, China). Subjects took blood pressure measurements twice at a 5-min interval from 7:00 to 9:00 a.m. on the physical examination day. Subjects were required to refrain from smoking or drinking tea or coffee within 30 min before the measurement and sit quietly for 15 min. Smoking was defined as smoking at least one cigarette per day in the past year; alcohol consumption was defined as alcohol consumption an average of 100 ml of white wine (alcohol content > 50%) per day for at least 1 year; physical exercise was defined as ≥ 3 exercise sessions/week for a duration of ≥ 30 min/session. Biochemical parameters, including total cholesterol (TC), triglycerides (TGs), high-density-lipoprotein cholesterol (HDL-C), low-density-lipoprotein cholesterol (LDL-C), and fasting blood glucose (FBG), were measured using an auto-analyzer (Hitachi 747; Hitachi, Tokyo, Japan) at the central laboratory at Kailuan General Hospital. baPWV data were collected using a BP-203RPE III networked arteriosclerosis detection device (Omron Health Medical Co., Ltd.). Hypertension was defined as systolic blood pressure (SBP) ≥ 140 mmHg and/or diastolic blood pressure (DBP) ≥ 90 mmHg or SBP < 140 mmHg and DBP < 90 mmHg with use of antihypertensive drugs. Diabetes was defined as fasting blood glucose (FBG) ≥ 7.0 mmol/L and FBG < 7.0 mmol/L with use of hypoglycemic agents or a history of diabetes. Dyslipidemia was defined as TC > 5.0 mmol/L or LDL-C > 3.0 mmol/L or TGs > 1.7 mmol/L or male HDL-C < 1.0 mmol/L. According to the Chinese Guidelines for the Control and Prevention of Overweight and Obesity in Adults, subjects were divided into two groups according to BMI (normal group: BMI < 24 kg/m^2^; overweight: BMI ≥ 24 kg/m^2^). According to the age segmentation criteria proposed by the United Nations World Health Organization, those aged < 45 years were the young subgroup and those aged ≥ 45 years were the non-young subgroup.

### Measurement of Noise in the Workplace

Measurement of noise in the workplace was carried out by Center for Disease Control and Prevention staff according to the technical requirements of GBZ/T189.8-2007 “Measurement of physical factors in the workplace—Part 8: Noise” (GBZ, 2007). The content includes on-site investigation before measurement, preparation of measuring instruments, setting of measuring points, on-site noise measurement, and measurement sound level calculation. Occupational noise exposure was defined as 8 h or equivalent of continuous sound level > 90 decibels (dB). Among the participants, 28.9% (3,215/11,141) were judged to have noise exposure.

### Assessment of Brachial Ankle Pulse Wave Velocity

The temperature of the examination room was kept between 22 and 25°C. Subjects were required to avoid smoking and rest for at least 5 min before the measurement. At the beginning of the test, subjects remained quiet, reclining on a bed without a pillow, palms up on both sides of the body. Upper arms and lower limbs were attached to the blood pressure cuffs. The marks on the upper arm sleeve balloon were located at the brachial artery; the lower cuffs of the arm sleeve were 2–3 cm away from the stripes of chelidon. The lower limb sleeve balloon marks were located on the inner side of the lower limbs; the lower cuffs were 1–2 cm away from the lower limb sleeves. The heart sound acquisition device was placed in the anterior cardiac area, and electrocardiograph collection devices were placed on the left and right wrists. The baPWV of each subject was measured twice, and the second measurement was taken as the final result. According to the criteria of the American College of Cardiology Medical Science Report, a baPWV value of < 14.0 m/s is typical for the peripheral artery and a baPWV value ≥ 14.0 m/s indicates peripheral arteriosclerosis (Yoshioka et al., 2010). In this study, the higher value of the left or right side baPWV was used for analyses.

### Assessment of Pure-Tone Average Hearing Threshold

Subjects remained in a quiet environment away from noise for a period of time, and the otologist checked and cleared the subjects’ ears. Subjects entered the sound-proof room and removed any glasses, headwear, or hearing aids. The otologist helped subjects put on earphones, and the subjects sat comfortably at least 30 s before the test. Subjects were asked not to touch the earphones and avoided unnecessary activities that could make extraneous noise. A Danish Metzda XETA clinical diagnostic audiometer (S/N: 961482) was used to measure pure-tone hearing threshold (0.5, 1, 2, and 4 kHz), and the otologist selected the ear test condition, sensor, unmasked/masked, and type of test on the device. First, using 1 kHz as the first pure frequency, the otologist sent a pure tone signal 20 dB below the audible hearing level; if there was no response, this was increased at 5-dB increments until a reaction was generated. If there was a reaction, the signal was reduced 5 dB, and the 5-dB hearing level increase experiment was repeated until two of the two or three minimum response hearing levels were the same. The same lowest audible hearing level was the frequency hearing level. The measurement of other frequencies was initiated near the hearing threshold of 1-kHz frequency (i.e., 0.5, 2, and 4 kHz). If there was a reaction, the signal was reduced by 5 dB; if there was no reaction, the signal was increased 5 dB and maintained 5–10 s until a reaction was generated. Because the frequencies of 0.5, 1, and 2 kHz account for 70% of language intelligibility, WHO issued a classification standard in 1997 adding another hearing threshold of 4 kHz to fully consider the higher-frequency HI of deaf people. PTA is the maximum value (i.e., worse) of the average hearing thresholds of the left or right ear at 0.5, 1, 2, and 4 kHz (in dB) (Friedland et al., 2009). Normal hearing is defined as PTA ≤ 25 dB, and HI is defined as PTA > 25 dB.

### Statistical Analyses

Data regarding lifestyle behaviors, physical examinations, baPWV, PTA, and biochemical parameters in this study were derived from the fifth follow-up from 2014 to 2015. Moreover, the fourth physical examination data from 2012 to 2013 were also used to replace missing values in the fifth follow-up data. One-way ANOVA and χ^2^ trend tests were used to calculate the significance of differences between groups in measurement or count data. Multivariate linear regression was used to calculate the quantitative relationship between baPWV and the PTA. In model 1, PTA was defined as the dependent variable, and baPWV was defined as an independent variable; model 2: age adjustment was added to model 1; model 3: SBP, FBG, TC, BMI, smoking, alcohol consumption, physical exercise, and noise exposure were adjusted and added to model 2. Multivariate logistic regression analysis was used to calculate the adjusted odds ratio (aOR) of baPWV for HI. In model 1, HI was defined as the dependent variable, and baPWV quartiles were defined as the independent variables (using the first quartile as the control group). In model 2, age was adjusted and added to model 1; model 3: BMI, smoking, alcohol consumption, physical exercise, hypertension, diabetes, dyslipidemia, and noise exposure were adjusted and added to model 2. Model 4: on the basis of model 3, the interactions between baPWV quartiles and age, hypertension, diabetes, and dyslipidemia were corrected, respectively. Multivariate logistic regression sensitivity analysis was used to calculate the aOR of baPWV for HI after removing subjects with noise exposure, hypertension, diabetes, and dyslipidemia, respectively, and was adjusted for age, BMI, smoking, alcohol consumption, physical exercise, hypertension, diabetes, dyslipidemia, and noise exposure. Model 1: removal of noise-exposed subjects (927 subjects); model 2: removal of the hypertensive population (284 subjects); model 3: removal of the diabetic population (61 subjects); model 4: removal of the dyslipidemia population (5,523 subjects). All analyses were conducted using SAS 9.3 (SAS Institute, Inc., Cary, NC, United States). Two-sided *p*-value < 0.05 was considered statistically significant.

### Ethics Statement

The Kailuan Study (registration no.: ChiCTR-TNC-11001489) was a survey and intervention study of cardiovascular disease risk factors based on a functional community population. The present study complied with the Declaration of Helsinki, in that the Kailuan Medical Group Ethics Committee approved the research protocol, and informed consent was obtained from all subjects.

## Results

### Characteristics of Subjects in Brachial Ankle Pulse Wave Velocity Quartile Groups

A total of 11,141 male subjects had a complete physical examination and biochemical data without head trauma or stroke history. The age range was 18–65 years, with mean age of 43.3 ± 8.9 years, and the average baPWV was 14.62 ± 2.56 m/s. Subjects were grouped into four subgroups according to baPWV quartile. First group (2,782 cases): baPWV ≤ 12.88 m/s; second group (2,778 cases): 12.88 m/s < baPWV ≤ 14.17 m/s; third group (2,797 cases): 14.17 m/s < baPWV ≤ 15.82 m/s; fourth group (2,787 cases): baPWV > 15.82 m/s. Age, SBP, DBP, BMI, TC, HDL-C, and FBG increased significantly as the baPWV quartile group increased (*p* < 0.05 for trend). Similarly, the proportions of subjects reporting smoking, alcohol consumption, hypertension, and diabetes increased significantly as the baPWV quartile group increased (*p* < 0.05 for trend). However, the proportion of participants reporting noise exposure decreased significantly as the baPWV quartile group increased (*p* < 0.05 for trend) (Table 1).

**TABLE 1:** Comparison of physical examination data, PTA, and HI detection rates for each baPWV quartile group.

Variable	First quartile group	Second quartile group	Third quartile group	Fourth quartile group	*P*-value
	*n* = 2,782	*n* = 2,778	*n* = 2,794	*n* = 2,787	
Age, years	38.36 ± 9.17	42.18 ± 8.43	44.60 ± 7.90	47.90 ± 7.10	<0.001
SBP, mmHg	121.64 ± 12.18	126.85 ± 12.51	131.67 ± 13.62	139.24 ± 15.59	<0.001
DBP, mmHg	76.59 ± 8.85	80.49 ± 8.94	83.49 ± 9.34	87.97 ± 9.92	<0.001
BMI, kg/m^2^	24.85 ± 3.73	25.06 ± 3.35	25.25 ± 3.20	25.38 ± 5.53	<0.001
TC, mmol/L	4.45 ± 1.38	4.75 ± 1.43	4.91 ± 1.53	5.01 ± 1.53	<0.001
HDL-C, mmol/L	1.34 ± 0.37	1.36 ± 0.58	1.38 ± 0.60	1.40 ± 0.56	<0.001
FBG, mmol/L	5.21 ± 1.11	5.36 ± 1.09	5.56 ± 1.39	5.98 ± 2.01	<0.001
Smoking, n (%)	1,278 (55.3)	1,352 (57.3)	1,360 (58.4)	1,383 (60.9)	<0.001
Alcohol consumption, n (%)	158 (5.7)	168 (6.0)	196 (7.0)	313 (11.2)	<0.001
Physical exercise, n (%)	122 (4.4)	129 (4.6)	128 (4.6)	135 (4.8)	0.46
Hypertension, n (%)	284 (10.2)	546 (19.7)	919 (32.9)	1,511 (54.2)	<0.001
Diabetes, n (%)	61 (2.2)	124 (4.5)	205 (7.3)	395 (14.2)	<0.001
Noise exposure, n (%)	927 (34.3)	759 (28.2)	793 (29.1)	736 (27.4)	<0.001
PTA, dBnHL	18.95 ± 8.27	20.12 ± 9.59	20.58 ± 10.46	22.51 ± 12.50	<0.001
HI, n (%)	284 (10.2)	405 (14.6)	476 (17.0)	656 (23.5)	<0.001

*SBP, systolic blood pressure; DBP, diastolic blood pressure; BMI, body mass index; TC, total cholesterol; HDL-C, high-density-lipoprotein cholesterol; FBG, fasting blood glucose; PTA, pure-tone average hearing threshold. The Cochran–Armitage trend test determined the p-value for a trend of categorical data changing in different subgroups, and the p-value for a trend of quantitative data changing in different subgroups was determined by linear regression analysis.*

### Pure-Tone Average Hearing Threshold and Hearing Impairment Detection Rates Among Subjects in Brachial Ankle Pulse Wave Velocity Quartile Groups

Among the 11,141 subjects, the average PTA was 20.54 ± 10.40 dB, and the detection rate of HI was 1,821/11,141 (16.3%). With the increase in baPWV quartile group, the PTA increased from 18.95 ± 8.27 dB to 22.51 ± 12.50 dB, and the detection rate of HI from the first quartile group to the fourth quartile group was 10.2 and 14.6%, and 17.0 and 23.5%, respectively (*p* < 0.05 for trend) (Table 1).

After multivariate linear regression analysis, it was found that for every 100 m/s increase in baPWV, the PTA increased by 13 dB (95% CI: 4–23) (Supplementary Table 1).

### Multivariate Logistic Regression Analysis of Brachial Ankle Pulse Wave Velocity Quartile Groups for Hearing Impairment

The aORs of HI comparing the first quartile group from the second to the fourth quartile groups were 1.16 (0.96–1.41), 1.11 (0.92–1.35), and 1.33 (1.10–1.62), respectively, after adjustment for age, BMI, smoking, alcohol consumption, physical exercise, hypertension, diabetes, dyslipidemia, and noise exposure. The interaction effect between baPWV quartile group and age on HI was statistically significant (*p* = 0.005), whereas the interaction effects between baPWV quartile groups and hypertension, diabetes, and dyslipidemia were not statistically significant (Table 2).

**TABLE 2:** Multivariate logistic regression analysis of baPWV quartile groups for HI.

Model	Factor	*B*-value	SE	Wald	*P*-value	OR	95% CI
Model 1	First quartile group					1.00	
	Second quartile group	0.41	0.08	24.23	<0.001	1.50	1.28–1.77
	Third quartile group	0.59	0.08	54.16	<0.001	1.81	1.54–2.11
	Fourth quartile group	1.00	0.77	167.74	<0.001	2.71	2.33–3.15
Model 2	First quartile group					1.00	
	Second quartile group	0.15	0.09	2.77	0.096	1.16	0.97–1.37
	Third quartile group	0.12	0.09	1.97	0.160	1.13	0.95–1.33
	Fourth quartile group	0.27	0.08	10.75	0.001	1.31	1.12–1.54
Model 3	First quartile group					1.00	
	Second quartile group	0.15	0.10	2.25	0.134	1.16	0.96–1.41
	Third quartile group	0.10	0.10	1.13	0.289	1.11	0.92–1.35
	Fourth quartile group	0.29	0.10	8.30	0.004	1.33	1.10–1.62
Model 4	baPWV × age	−0.21	0.08	7.80	0.005	0.81	0.70–0.94
	baPWV × hypertension	0.03	0.07	0.24	0.627	1.03	0.91–1.18
	baPWV × diabetes	0.06	0.11	0.34	0.557	1.06	0.86–1.31
	baPWV × dyslipidemia	0.10	0.06	2.31	0.129	1.10	0.97–1.24

*Model 1: HI was defined as the dependent variable, and baPWV quartiles were defined as the independent variables (using the first quartile as the control group), and logistic regression analysis was performed. Model 2: age was adjusted and added to model 1. Model 3: body mass index, smoking, alcohol consumption, physical exercise, hypertension, diabetes, dyslipidemia, and noise exposure were adjusted and added to model 2. Model 4: on the basis of model 3, the interactions between baPWV quartiles and age, hypertension, diabetes, and dyslipidemia, respectively, were corrected.*

### Multivariate Logistic Regression Sensitivity Analysis of Brachial Ankle Pulse Wave Velocity Quartile Groups for Hearing Impairment

Comparing the first quartile group with the second, third, and fourth quartile groups, the aOR values were 1.28 (1.02–1.60), 1.29 (1.02–1.63), 1.30 (1.06–1.59), and 1.20 (0.83–1.73), respectively, after removing subjects reporting noise exposure, hypertension, diabetes, and dyslipidemia, respectively, and adjusted by age, BMI, smoking, alcohol consumption, physical exercise, hypertension, diabetes, dyslipidemia, and noise exposure (Table 3).

**TABLE 3:** Multivariate logistic regression sensitivity analysis of baPWV quartile groups for HI.

	Factor	*B*-value	SE	Wald	*P*-value	OR	95% CI
Model 1	First quartile group					1.00	
	Second quartile group	0.13	0.11	1.23	0.267	1.14	0.91–1.42
	Third quartile group	0.09	0.11	0.64	0.425	1.10	0.88–1.37
	Fourth quartile group	0.25	0.11	4.71	0.030	1.28	1.02–1.60
Model 2	First quartile group					1.00	
	Second quartile group	0.17	0.11	2.32	0.128	1.18	0.95–1.46
	Third quartile group	0.18	0.11	2.71	0.100	1.20	0.97–1.49
	Fourth quartile group	0.25	0.12	4.44	0.035	1.29	1.02–1.63
Model 3	First quartile group					1.00	
	Second quartile group	0.13	0.10	1.62	0.203	1.14	0.93–1.39
	Third quartile group	0.07	0.10	0.48	0.489	1.07	0.88–1.31
	Fourth quartile group	0.26	0.10	6.38	0.012	1.30	1.06–1.59
Model 4	First quartile group					1.00	
	Second quartile group	0.27	0.16	2.83	0.092	1.32	0.96–1.81
	Third quartile group	<0.001	0.17	<0.001	0.998	1.00	0.71–1.41
	Fourth quartile group	0.18	0.19	0.95	0.329	1.20	0.83–1.73

*Model 1: removed noise-exposed subjects. Model 2: removed the hypertensive population. Model 3: removed the diabetic population. Model 4: removed dyslipidemia population.*

### Multivariate Logistic Regression Analysis of Brachial Ankle Pulse Wave Velocity Quartile Group for Hearing Impairment in Different Age Subgroups

Among the young subgroup (5,478 subjects; age range 22–44 years; mean age 35.6 ± 5.5 years), the aORs of HI in the second to fourth quartile groups compared with the first quartile group were 1.55 (1.04–2.31), 1.79 (1.18–2.72), and 2.65 (1.68–4.19). Among the non-young subgroup (5,663 subjects; age range 45–65 years; mean age 50.7 ± 3.7 years), the aORs of HI in the second to fourth quartile groups were 1.04 (0.83–1.30), 0.96 (0.77–1.20), and 1.13 (0.91–1.40) (Table 4). Moreover, multivariate linear regression analysis indicated that the PTA increased by 18 dB (95% CI: 10–27) with every 100 m/s increase in baPWV in the young subgroup, and PTA increased by 10 dB (95% CI: −7 to 26) in the non-young subgroup (Supplementary Table 2).

**TABLE 4:** Bivariate and multivariate logistic regression analysis of HI in different age groups.

Model	Factor	Age < 45 (*n* = 5,478)	Age ≥ 45 (*n* = 5,663)
		OR (95% CI)	OR (95% CI)
Model 1	First quartile group	1	1
	Second quartile group	1.46 (1.01–2.11)	1.06 (0.87–1.29)
	Third quartile group	1.74 (1.20–2.52)	0.99 (0.82–1.20)
	Fourth quartile group	2.29 (1.54–3.42)	1.15 (0.96–1.38)
Model 2	First quartile group	1	1
	Second quartile group	1.55 (1.04–2.31)	1.04 (0.83–1.30)
	Third quartile group	1.79 (1.18–2.72)	0.96 (0.77–1.20)
	Fourth quartile group	2.65 (1.68–4.19)	1.13 (0.91–1.40)
P for interaction		<0.001	

*Model 1: bivariate logistic regression analysis was conducted after age stratification (young subgroup: age < 45, non-young subgroup: age ≥ 45), with HI defined as the dependent variable and baPWV defined as the independent variable (the first quartile was the control group). Model 2: BMI, smoking, alcohol consumption, physical exercise, hypertension, diabetes, dyslipidemia, and noise exposure were adjusted.*

## Discussion

Our survey showed that the detection rate of HI significantly increased with increasing baPWV quartile groups, and for every 100 m/s increase in baPWV, the PTA increased by 13 dB. This relationship was robust after adjustment for demographic and physical condition confounders. It is worth noting that this relationship seemed inconsistent between the young and non-young subgroups. The PTA increment increased to 18 dB for every 100 m/s increase in baPWV in the young subgroup. However, this relationship was not statistically significant in the non-young subgroup.

To our knowledge, this is the first study demonstrating a reliable quantitative relationship between baPWV and PTA or HI detection rate using such a large sample size (11,141 subjects) in China or elsewhere in the world. We found that the HI detection rate increased significantly with increasing baPWV quartile groups, and this relationship was robust after adjustment for various demographic and physical condition confounders (aOR: 1.33, 95% CI: 1.10–1.62, fourth vs. first quartile groups) (Table 3). Moreover, the PTA increased by 13 dB (95% CI: 4–23) based on multivariate linear regression analysis (Supplementary Table 1). Some studies have conducted similar surveys with results similar to ours. Helzner et al. (2011) found that poor hearing sensitivity was associated with high BMI, high resting heart rate, and fast PWV; however, the subjects (70–79 years; mean age 77.5 years; 48% men; Medicare beneficiaries) in their study were elderly. Fischer et al. (2015) reported that subclinical atherosclerosis was associated with HI risk in a large cohort of middle-aged participants (35–64 years; men and women; community-based cohort). In contrast to our study, however, atherosclerosis was assessed by the mean carotid intima–media thickness (Fischer et al., 2015). Chung et al. (2016) found that higher baPWV was associated with the development and severity of sudden sensorineural HI, and baPWV was correlated with initial hearing threshold. However, only 108 subjects were included in that study (22–74 years; 54 sudden sensorineural HI patients and 54 age- and sex-matched controls; 51.1 ± 13.8 years old; men and women), and the calculation procedure did not consider adjustment for all potential confounders affecting HI (Chung et al., 2016). Yoshioka et al. (2010) found that arterial sclerosis exacerbates the deleterious effects of noise on hearing (773 subjects; 40–83 years; men; 60.3 ± 11.6 years old; 28% were exposed to occupational noise). Erkan et al. (2015) (265 patients underwent coronary angiography for symptoms suggesting ischemic heart disease and who had ischemia detected; 61.5 ± 13.0 years; 55.1% men and 44.9% women) found a statistically significant positive correlation between the degree of HI and Gensini score (*via* coronary angiography), and this correlation remained significant after adjustment for other risk factors. A statistically significant negative correlation between Gensini score and speech discrimination score has also been reported (Erkan et al., 2015). However, as the sample sizes were small in these studies, and the subjects were even older than in ours or the calculation procedures did not consider demographic and physical condition confounders, the quantitative relationships between atherosclerosis and HI in those studies lack consistency and reliability compared with our study (Yoshioka et al., 2010; Helzner et al., 2011; Erkan et al., 2015; Fischer et al., 2015; Chung et al., 2016).

To further explore the reliability of our results, we conducted an interaction analysis between various known risk factors shared by atherosclerosis and HI [e.g., age (Lee et al., 2005; Pratt et al., 2009), hypertension (Przewozny et al., 2015), diabetes (Akinpelu et al., 2014), and dyslipidemia (Chang et al., 2014)] and baPWV quartile groups for HI, as they increased significantly as the baPWV quartile group increased (Table 1). The result was unexpected in that the interaction effects of hypertension, diabetes, or dyslipidemia and baPWV for HI were not statistically significant (Table 3, model 4). However, the interaction effect of age and baPWV might have a negative impact on HI (aOR: 0.81, 95% CI: 0.70–0.94) (Table 3, model 4), which suggests that the effect of baPWV on HI decreases as age increases. Based on this assumption, to control for the effect of age on the relationship between baPWV and HI, we divided the subjects into young and non-young subgroups (not old persons, as the upper cut-off was 65 years) based on a cut-off age of 45 years. We then calculated the respective relationships between baPWV and HI or PTA in young and non-young subgroups. These results were also unexpected, in that the relationship was not consistent between the young and non-young subgroups. In the young subgroup, the aOR of baPWV for HI increased to 2.65 (95% CI: 1.68–4.19, fourth vs. first quartile groups) (Table 4). Moreover, the PTA increment increased to 18 dB (95% CI: 10–27) for every 100 m/s increase in baPWV (Supplementary Table 2). However, the relationship was not statistically significant in the non-young subgroup (aOR: 1.13, 95% CI: 0.91–1.40), suggesting that prediction of HI using baPWV is not reliable for middle-aged people (Table 4 and Supplementary Table 2).

To our knowledge, few studies have compared the feasibility of using baPWV to predict HI between young and non-young people. Furthermore, studies targeting older people have not shown consistent results. Helzner et al. (2011) surveyed 2,049 subjects of average age 77.5 years and found no linear correlation between cfPWV and PTA, consistent with our study. However, Erkan et al. (2015) conducted a study involving 265 subjects aged 61.5 ± 13.0 years and reported a linear correlation between Gensini score for assessing the severity of coronary artery stenosis and PTA. The available evidence thus indicates that atherosclerosis is not a reliable independent risk factor for HI among non-young people. In these individuals, degeneration associated with aging might be the dominant risk factor for HI, and the effects of other risk factors, including atherosclerosis, might be overshadowed by aging, so that the statistical weight of the impact of atherosclerosis declines (Lee et al., 2005; Pratt et al., 2009). Furthermore, the prevalence of hypertension, diabetes, dyslipidemia, smoking, and alcohol consumption is generally higher among middle-aged people, and these systemic diseases or unhealthy lifestyle habits have been shown to have a potential pathophysiological association with atherosclerosis and therefore could directly induce HI *via* microangiopathy and neuropathy (Wood, 2001; Pearson, 2002; Bisoendial et al., 2003; Hansson, 2005; Liu et al., 2007). During the statistical analysis, all of these risk factors were considered as confounders and adjusted by multivariate logistic/linear regression to calculate the independent effect of baPWV for HI, which would unavoidably weaken the correlation between baPWV and HI (the linear relationship became statistically insignificant after adjustment for such confounders in model 2) (Supplementary Table 2). Our results indicate that predicting HI using baPWV is more applicable for younger people compared with middle-aged people, as without the interference of serious systemic diseases, it was easier to quantify the possible effect of subclinical atherosclerosis on HI in younger individuals. More importantly, if screening baPWV is integrated into primary health examinations, more young people will pay attention to their vascular health and change their lifestyle to avoid HI.

To evaluate the weights of the effect of various known risk factors on HI, we conducted a multivariate logistic regression sensitivity analysis by removing the subjects with such risk factors from the dataset. Numerous studies have demonstrated the physiopathological mechanism of noise exposure (Saunders et al., 1985; Henderson and Hamernik, 1986), diabetes (Tsilibary, 2003; Carlson et al., 2004; Gibbons and Shaw, 2012), and hypertension (Przewozny et al., 2015) on HI. However, the relationship between baPWV and HI seemed to diminish after these risk factors were eliminated. Unlike noise exposure, diabetes, and hypertension, the relationship between baPWV and HI became statistically insignificant after removal of dyslipidemia subjects (Table 4). This result might indicate that dyslipidemia played a dominant role in the relationship between atherosclerosis and HI in our study subjects. In other words, dyslipidemia could be the most immediate factor in the pathogenesis of atherosclerosis compared with other known risk factors (Hansson, 2005). However, atherosclerosis is a complex, multi-etiological disease. Based on our results, SBP, DBP, BMI, TC, HDL-C, FBG, smoking, and alcohol consumption increased significantly as the baPWV quartile group increased (Table 1). Thus, to avoid subclinical atherosclerosis, subjects need the necessary treatment to control their blood lipids and blood pressure and should maintain a healthy lifestyle and balanced diet.

Our study still had some limitations. The subjects were all males, and all subjects were employees or retirees of a single coal mining company (Kailuan Group Company) in Tangshan City, China; therefore, the quantitative relationship between baPWV and HI might only apply to males in northern China. Also, the subjects in the Kailuan cohort were exposed to occupational hazards (such as noise). Hence, the results found in this study may not be generalized beyond the population with noise exposure. Moreover, we found that the quantitative relationship between baPWV and HI was inconsistent between the young and non-young subgroups. We deduced that applying this quantitative relationship might require more specific qualifications (e.g., gender, age, and region). More baPWV and PTA measurement surveys are needed in other parts of China to better define the parameters. Moreover, theoretically, arteriosclerosis initially affects lower frequencies (0.25, 0.5, and 1 KHz) (Liew et al., 2007); however, our study cannot further explore this characteristic because the subjects’ audiometric assessment in Kailuan cohort was conducted by Occupational Disease Control Center of the Kailuan Group. The otologists measured the PTA according to the standard clinical practice, which includes the frequencies of 0.5, 1, 2, and 4 kHz. These frequencies are able to account for almost all of the language intelligibility, so that the frequencies of 250 Hz or below were not being considered to measure. We will add the frequencies of 250 Hz or below in the next follow-up visit.

## Conclusion

Subclinical atherosclerosis and mild HI lack apparent symptoms and thus easily go unnoticed by patients, especially younger individuals with an unhealthy lifestyle or diet. However, atherosclerosis and HI still cannot be completely cured, and as the diseases continue to advance, they can seriously affect quality of life or even threaten a patient’s life. Our large sample size, cross-sectional survey explores the quantitative relationship between baPWV and HI. Although this relationship is not universally applicable or consistent among different populations and the subjects in the Kailuan cohort were exposed to occupational hazards (such as noise) could not be representative of the general population, it is still enlightening that people should pay more attention to their blood vessels’ condition, to avoid atherosclerosis and its complications.

## Data Availability Statement

The raw data supporting the conclusions of this article will be made available by the authors, without undue reservation.

## Ethics Statement

The studies involving human participants were reviewed and approved by the Kailuan Medical Group Ethics Committee. The patients/participants provided their written informed consent to participate in this study.

## Author Contributions

WW and SW contributed to conception and design of experiments. WW, SW, and CR carried out the study and experiments. XM and SC analyzed the data. XM, WW, and CR wrote and revised the article. All authors reviewed the article.

## Conflict of Interest

The authors declare that the research was conducted in the absence of any commercial or financial relationships that could be construed as a potential conflict of interest.

## Publisher’s Note

All claims expressed in this article are solely those of the authors and do not necessarily represent those of their affiliated organizations, or those of the publisher, the editors and the reviewers. Any product that may be evaluated in this article, or claim that may be made by its manufacturer, is not guaranteed or endorsed by the publisher.

## Abbreviations

HI: hearing impairment
PWV: pulse wave velocity
cfPWV: carotid to femoral PWV
baPWV: brachial ankle PWV
PTA: pure-tone average hearing threshold
BMI: body mass index
TC: total cholesterol
TG: triglyceride
HDL-C: high-density-lipoprotein cholesterol
LDL-C: low-density-lipoprotein cholesterol
FBG: fasting blood glucose
SBP: systolic blood pressure
DBP: diastolic blood pressure
OR: odds ratio.
