# Supplementary material for: Subclinical Atherosclerosis Could Increase the Risk of Hearing Impairment in Males: A Community-Based Cross-Sectional Survey of the Kailuan Study
Source: Front Neurosci. 2022 Apr 25;16:813628. doi: 10.3389/fnins.2022.813628 (PMC9082793; doi:10.3389/fnins.2022.813628)
Supplement: Supplementary file 1 [file Table_1.docx]

Supplemental Table 1. Multivariate linear regression analysis according to PTA

| Model | Factor | B Value | S.E. | Standard B Value | R^2^ | P | B (95% CI) | VIF |
| --- | --- | --- | --- | --- | --- | --- | --- | --- |
| Model 1 | baPWV | 0.53 | 0.04 | 0.13 | 0.017 | <0.001 | 0.45 to 0.60 | 1 |
| Model 2 | baPWV | 0.14 | 0.04 | 0.04 | 0.094 | <0.001 | 0.07 to 0.22 | 1.12 |
| Model 3 | baPWV | 0.13 | 0.05 | 0.03 | 0.098 | 0.007 | 0.04 to 0.23 | 1.41 |

*Note* Model 1: PTA was defined as the dependent variable, and baPWV was defined as an independent variable for multivariate linear regression analysis; Model 2: Age was adjusted and added to model 1; Model 3: systolic blood pressure, fasting blood glucose, total cholesterol, body mass index, smoking, alcohol consumption, physical exercise, and noise exposure were adjusted and added to model 2.

Supplemental Table 2. Multivariate linear regression analysis of the PTA in different age subgroups

| Model | Factor | Age < 45 (n=5,478) | P |  | Age ≥ 45 (n=5,663) | P |
| --- | --- | --- | --- | --- | --- | --- |
|  |  | B (95% CI) |  |  | B (95% CI) |  |
| Model 1 | baPWV | 0.16 (0.09 to 0.22) | <0.001 |  | 0.14 (0.02 to 0.26) | 0.026 |
| Model 2 | baPWV | 0.18 (0.10 to 0.27) | <0.001 |  | 0.10 (−0.07 to 0.26) | 0.250 |

*Note* Model 1: Multivariate linear regression analysis was conducted after age stratification (young subgroup: age <45, non-young subgroup: age ≥ 45), with PTA defined as the dependent variable and baPWV defined as the independent variable. Model 2: fasting blood glucose, total cholesterol, BMI, smoking, alcohol consumption, physical exercise, and noise exposure were adjusted.
